# Transcriptomic insights into mycorrhizal interactions with tomato root: a comparative study of short- and long-term post-inoculation responses

**DOI:** 10.3389/fgene.2024.1434761

**Published:** 2024-10-08

**Authors:** Mohamed Abdelsattar, Maali S. Soliman, Rasha A. Mohamed, Khaled H. Radwan, Mohamed M. El-Mahdy, Khaled H. Mousa, Shaimaa R. M. Khalil, Engy Osman, Hussien F. Alameldin, Ahmed Hussein, Sameh E. Hassanein, Naglaa A. Abdallah, Alsamman M. Alsamman, Omnia Osama

**Affiliations:** ^1^ Agricultural Genetic Engineering Research Institute (AGERI), Agricultural Research Center (ARC), Giza, Egypt; ^2^ Plant Pathology Research Institute, Agricultural Research Center, Giza, Egypt; ^3^ The Central Laboratory for Phytosanitary and Food Safety, United Integrated Laboratories, Barka, Oman; ^4^ National Biotechnology Network of Expertise, ASRT, Cairo, Egypt; ^5^ Department of Genetics, Faculty of Agriculture, Cairo University, Giza, Egypt; ^6^ Sugarbeet and Bean Research Unit, U.S. Department of Agriculture - Agriculture Research Service (USDA-ARS), East Lansing, MI, United States; ^7^ International Center for Agriculture Research in the Dry Areas (ICARD), Giza, Egypt

**Keywords:** arbuscular mycorrhiza, *Rhizophagus irregularis*, *Solanum lycopersicum* L., RNA-seq, symbiosis

## Abstract

**Background:**

Arbuscular mycorrhiza (AM) refers to a symbiotic association between plant roots and fungi that enhances the uptake of mineral nutrients from the soil and enables the plant to tolerate abiotic and biotic stresses. Although previously reported RNA-seq analyses have identified large numbers of AM-responsive genes in model plants, such as *Solanum lycopersicum* L., further studies are underway to comprehensively understand the complex interactions between plant roots and AM, especially in terms of the short- and long-term responses after inoculation.

**Results:**

Herein, we used RNA-seq technology to obtain the transcriptomes of tomato roots inoculated with the fungus *Rhizophagus irregularis* at 7 and 30 days post inoculation (dpi). Of the 1,019 differentially expressed genes (DEGs) in tomato roots, 635 genes showed differential expressions between mycorrhizal and non-mycorrhizal associations at the two time points. The number of upregulated DEGs far exceeded the number of downregulated ones at 7 dpi, and this difference decreased at 30 dpi. Several notable genes were particularly involved in the plant defense, plant growth and development, ion transport, and biological processes, namely, *GABAT*, *AGP*, *POD*, *NQO1*, *MT4*, *MTA*, and *AROGP3*. In addition, the Kyoto encyclopedia of genes and genomes pathway enrichment analysis revealed that some of the genes were involved in different pathways, including those of ascorbic acid (*AFRR*, *GME1*, and *APX*), metabolism (*CYP*, *GAPC2*, and *CAM2*), and sterols (*CYC1* and *HMGR*), as well as genes related to cell division and cell cycle (*CDKB2* and *PCNA*).

**Conclusion:**

These findings provide valuable new data on AM-responsive genes in tomato roots at both short- and long-term postinoculation stages, enabling the deciphering of biological interactions between tomato roots and symbiotic fungi.

## 1 Introduction

Profound changes can be observed in the metabolomes of plants, such as tomatoes, from mycorrhizal interactions (Rivero et al., 2015). In such instances, it is also noted that under certain conditions, arbuscular mycorrhizal fungi (AMF) as well as non-cultivated plant genes are expressed in tomato roots under the affected mycorrhiza (Ruzicka et al., 2013). Mycorrhizal interactions are also known to affect tissues that are far away from the roots; in tomato plants, their influence also extends to fruit metabolism (Zouari et al., 2014). This clearly shows the systemic effects of mycorrhizal interactions on the physiological functions of plants. Many of the molecular mechanisms involved in mycorrhizal symbiosis are only now being uncovered slowly, but sucrose transport has been found to be regulated by various genes as well as oxylipin metabolism and gene expression (Bitterlich et al., 2014; León Morcillo et al., 2012). Scientists have devoted extensive efforts to determining how mycorrhizal symbiosis works in plants, but this area of research has remained underinvestigated. The dynamic interactions between mycorrhizal symbiosis and other environmental factors, such as nitrogen availability, have been revealed through the linked gene expression profiles of mycorrhizal tomato roots grown in different nutrient environments (Ruzicka et al., 2010; Ruzicka et al., 2012). In addition, research indicates that mycorrhizal fungi enhance the defense mechanisms against pathogens, such as nematodes, demonstrating the multifaceted role of AM symbiosis in plant health (Silva et al., 2022; Mahdy et al., 2008). Transcriptional profiling techniques such as microarray and RNA sequencing have been used for expression analyses of the interactions between arbuscular mycorrhiza (AM) and tomato plants in the roots, leaves, and fruits during the developmental stages (Schubert et al., 2020; Cervantes-Gámez et al., 2016; Zouari et al., 2014; Ullah et al., 2023). AMF have been shown to significantly alter gene expressions in the roots of various plant species (Hogekamp and Küster, 2013; Groten et al., 2015b; Casarrubias-Castillo et al., 2020). Interestingly, very few studies have reported the differentially expressed genes (DEGs) in AM-inoculated tomato roots using microarray analysis (Chialva et al., 2019; Dermatsev et al., 2010; Fiorilli et al., 2009) and RNA-seq analysis (Sugimura and Saito, 2017; Vangelisti et al., 2019; Zeng et al., 2023; Tominaga et al., 2022) by focusing specifically on the early and late stages of inoculation. AM have been shown to mediate various aspects of plant physiology, including lipid peroxidation regulation, reactive oxygen species (ROS) level control, and antioxidant enzyme accumulation. These interactions play crucial roles in enhancing plant defense mechanisms against oxidative stresses. Moreover, the key antioxidant enzymes and mechanisms include proanthocyanidins, flavonoids, ascorbic acid, superoxide dismutase (SOD), monodehydroascorbate reductase (MDAR), peroxidase (POX), and total antioxidant capacities (Hogekamp and Küster, 2013; Groten et al., 2015a; Guether et al., 2011). Notably, previous studies have shown that both short- and long-term colonization by AMF can transcriptionally promote a specific retrotransposon in the roots of the sunflower plant (Vangelisti et al., 2019). Additionally, post-transcriptional regulation has been shown to play a significant role in AM regulation in tomato roots (Zeng et al., 2023). By recognizing the interplay in these processes, we aim to provide a comprehensive picture of the tomato root transcriptome under mycorrhizal interactions. Through comparative analyses of the short- and long-term post-infection responses, we expect to unravel some of the complex regulatory networks and molecular pathways that maintain the symbiotic relationships between plants and fungi, thereby shedding light on the vast field of knowledge encompassing plant–mycorrhiza interactions.

## 2 Methodology

### 2.1 Plant material and greenhouse experiment

Tomato (*Solanum lycopersicum* L.) cultivar Heinz “Hz” was kindly provided by Heinz Company, Egypt, and was used as the experimental plant. The tomato seeds were first sterilized using sodium hypochlorite (NaOCl, 5% v/v) and washed three times with distilled water, followed by sowing in trays containing a sterilized mixture of peat, perlite, and sand (1:1:1 v/v/v). Four-week-old tomato seedlings were then transplanted in 1-L pots containing a mixture of sterilized loam soil, sand, and peat moss (1:1:1 v/v/v). The plantlets were grown in controlled conditions in a greenhouse under 14/10 h day/night cycles at 30.0 ± 5.0°C during the day/night, 56.0 ± 14.2% Relative Humidity (RV), and 20.0 klux.

### 2.2 Fungal culture

*Rhizophagus irregularis* (ON869380) was provided by the Plant Pathology Research Institute (PPATHRI), ARC, Giza, Egypt. *Rhizophagus irregularis* was propagated on Sudangrass (*Sorghum sudanese* trap plant) grown for 4 months (Bethlenfalvay et al., 1982). During transplantation, 10 g/pot (clay:sand at 1:2) of the respective AMF inoculum (230 spores/50 g) was added per plant, and ten pots (3 plants/pot per experiment) were used as the replicates. The pots were arranged in a completely randomized design and maintained in a greenhouse under 14/10 h day/night cycles at 30.0 ± 5.0°C during the day/night, 56.0 ± 14.2% RV, and 20.0 klux. Thirty days after sowing, the soil was infested with the *R. irregularis* inoculum at a concentration of 2.5% (w/w). Root samples were collected for analysis at two time points of 7 and 30 days post inoculation (dpi) and frozen immediately at −80°C.

### 2.3 Detecting AM colonization of roots

The tomato roots were cut into short segments (5–8 cm), placed in test tubes, and immersed in 10% KOH. The tubes were then placed in a water bath and boiled for 5 min to allow appropriate clearing. The roots segments were next rinsed a few times with tap water. Staining was performed with 10% ink–vinegar solution (Pelikan, Germany) prepared using pure white household vinegar. The roots were then destained by rinsing in vinegar for 20 min (Vierheilig et al., 1998) and examined under a light microscope at 20× (AX10, ZEISS Group, Germany).

### 2.4 Genomic DNA extraction

Tomato roots frozen in liquid nitrogen were ground, and the genomic DNA was extracted using the DNeasy Plant Mini Kit (Qiagen, Hilden, Germany) according to the manufacturer’s instructions. To isolate the DNA from the AM-inoculated roots, the QIAprep Spin Miniprep Kit (Qiagen, Hilden, Germany) was used according to the manufacturer’s instructions. The quality and quantity of genomic DNA were measured using the NanoDrop™ 2000 spectrophotometer (Thermo Fisher Scientific, Waltham, MA, United States).

### 2.5 *R. irregularis* detection

AMF colonization was assessed using quantitative polymerase chain reaction (qPCR) (Alkan et al., 2004). Briefly, *R. irregularis* was quantified based on the *Glomus intraradices* (Gi) AM gene, which was normalized to the plant gene (Actin) (Pirrello et al., 2006). The PCR amplifications were performed in a solution with a total volume of 20 µL containing 1 µL (10 ng/µL) of the diluted DNA, 10 µL of 2× BioEasy SYBR Green Master Mix (BIOER, Hangzhou, China), 1 µL (10 µM) of GiAM-F (5’-GCT CTG GTG CCG AAA GCT T-3’) and GiAM-R (5’-TAA CCC GTT CTA ACC TAT TGA CCA T-3’), actin-F (5’-TGT​CCC​TAT​TTA​CGA​GGG​TTA​TGC-3’), and actin-R (5’ CAG​TTA​AAT​CAC​GAC​CAG​CAA​GAT-3’). Furthermore, qPCRs were conducted using the SYBR Green Master Mix on the Mx3000P QPCR System (Agilent Technologies, Inc., Santa Clara, CA, United States) with a starting cycle at 95°C for 2 min, followed by 35 cycles each at 95°C for 5 s and at 60°C for 40 s. The GiAM gene signal from the AMF was normalized to the plant gene signal using Eq. (1):

$$\frac{\text{AMF gene}\left(\text{GiAM}\right)}{\text{plant gene}\left(\text{Actin}\right)}=\frac{E\text{\_}{\text{Actin}}^{Ct-\text{Actin}}}{E\text{\_}{\text{GiAM}}^{Ct-\text{GiAM}}}$$ (1)

where Ct is the mean value from three technical replicates, and E is the mean value from all reactions with a particular primer pair for each run (Bodenhausen et al., 2014).

### 2.6 RNA isolation and sequencing with data analysis

The selected roots that were previously identified using qPCR were sampled for RNA extraction as follows: whole roots of the non-inoculated (control) and AM-inoculated plant samples were rapidly ground manually in a mortar using liquid nitrogen. Subsequently, 100 µg of the total ground tissue was used for total RNA extraction, and three biological replicates were performed for each sample. The total RNA was extracted using TRIzol reagent (Invitrogen, United States) and treated with RQ1 DNase (Promega, United States) according to the manufacturer’s protocols. The quality control and quantification of the total RNA were conducted using an Agilent 2200 TapeStation bioanalyzer (Santa Clara, CA, United States). Three independent samples were prepared for each group. Library construction and RNA sequencing were conducted by researchers at the Beijing Genomics Institute (BGI) using the DNBSEQ high-throughput platform, which produced approximately 3.5 GB of data made up of 150-base-long paired reads per sample.

Trimmomatic tools have been previously used to remove adapter sequences and low-quality reads (Bolger et al., 2014). The paired-end clean (150 bp) reads were then aligned to the tomato reference genome GCF_000188115.4_*SL*3.0 using HISAT (Kim et al., 2015). The fragments per kilobase of transcripts per million mapped reads (FPKM) of each gene was calculated from its length and reads count mapped (Anders et al., 2010). Differential expression analysis was then performed using the DESeq2 R package. An adjusted *p*-value cutoff of <0.05 and absolute fold change ≥2 were used to identify the DEGs.

### 2.7 DEG analysis

Analysis of the DEGs between the infected groups and controls was performed using the DESeq2 package in the R programming language (http://www.r-project.org/) to identify the genes with significant differences in their expressions. Genes that had a false discovery rate (FDR) of ≤0.01 were categorized as significant DEGs, which were visualized with heatmaps created using the ggplot2 package. This visualization technique aids intuitive interpretation of the gene expression patterns, while facilitating the identification of important trends and clusters within the data. In addition, principal component analysis (PCA) was used to gain insights into the relationships and differences between the different stages; this analysis was performed using iDEP software (http://bioinformatics.sdstate.edu/idep96/). In addition, we used the online software VennDiagram (https://bioinformatics.psb.ugent.be/webtools/Venn/) to create Venn diagrams for comparative analysis during the different stages of the experiments. These diagrams facilitate exploration of the common and unique genes at different stages.

### 2.8 Gene ontology (GO) analysis and protein–protein interaction (PPI) network construction

The STRING online database (https://string-db.org/) was employed in the functional enrichment analysis of the DEGs. This comprehensive analysis involves mapping all target gene candidates to the GO terms, including biological processes (BPs), cellular components (CCs), and molecular functions (MFs), as well as the Kyoto encyclopedia of genes and genomes (KEGG) pathways. The PPI network of the DEGs was established using the STRING database to evaluate the functional associations among the proteins. The PPI network was visualized using Cytoscape (v3.10.2), with the nodes representing the proteins and edges representing the interactions.

## 3 Results

### 3.1 AM colonization of tomato roots

**FIGURE 1:**
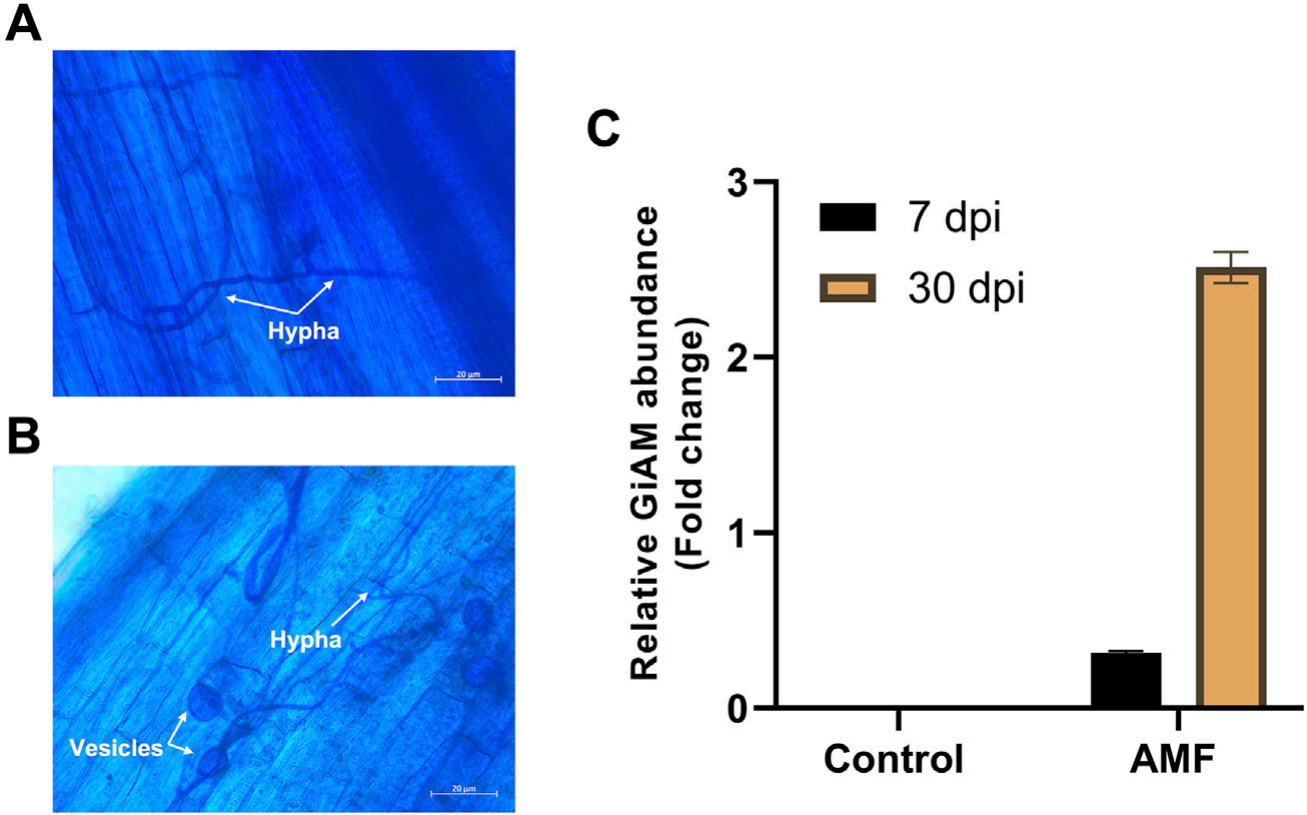
Tomato root colonization of arbuscular mycorrhiza (AM) observed under a light microscope at 20× magnification: **(A)** AM hyphal formation at 7 dpi, **(B)** AM hyphal and vesicle formations at 30 dpi, **(C)** quantifications of AM inoculation at 7 and 30 dpi. The data are presented as mean ± standard error of the mean (SEM) (n = 3 independent trials with 6–8 plants per treatment per trial).

The AMF inoculates established a symbiotic relationship with the tomato roots, and the tomato roots inoculated with AM showed clear occupation by the fungus. These developments were clearly visible at 7 and 30 dpi. At 7 dpi, hyphae were observed in the tomato roots, indicating root colonization (Figure 1A); at 30 dpi, hyphae and vesicles were observed (Figure 1B). This shows that colonization is a perpetual process even under long-term colonization conditions. Tomato roots inoculated with AM were subjected to qPCR analyses at 7 and 30 dpi to confirm sufficient colonization of *R. irregularis* in the tomato roots. The AMF root colonization with GiAM as the specific gene showed that AM colonization was 2.5- and 0.3-fold higher at 30 and 7 dpi, respectively. In addition, no AMF were detected in the non-inoculated tomato roots, confirming the absence of cross-contamination and AM colonization at 7 and 30 dpi (Figure 1C). This confirms that AM colonization increases under long-term conditions.

### 3.2 DEGs

**FIGURE 2:**
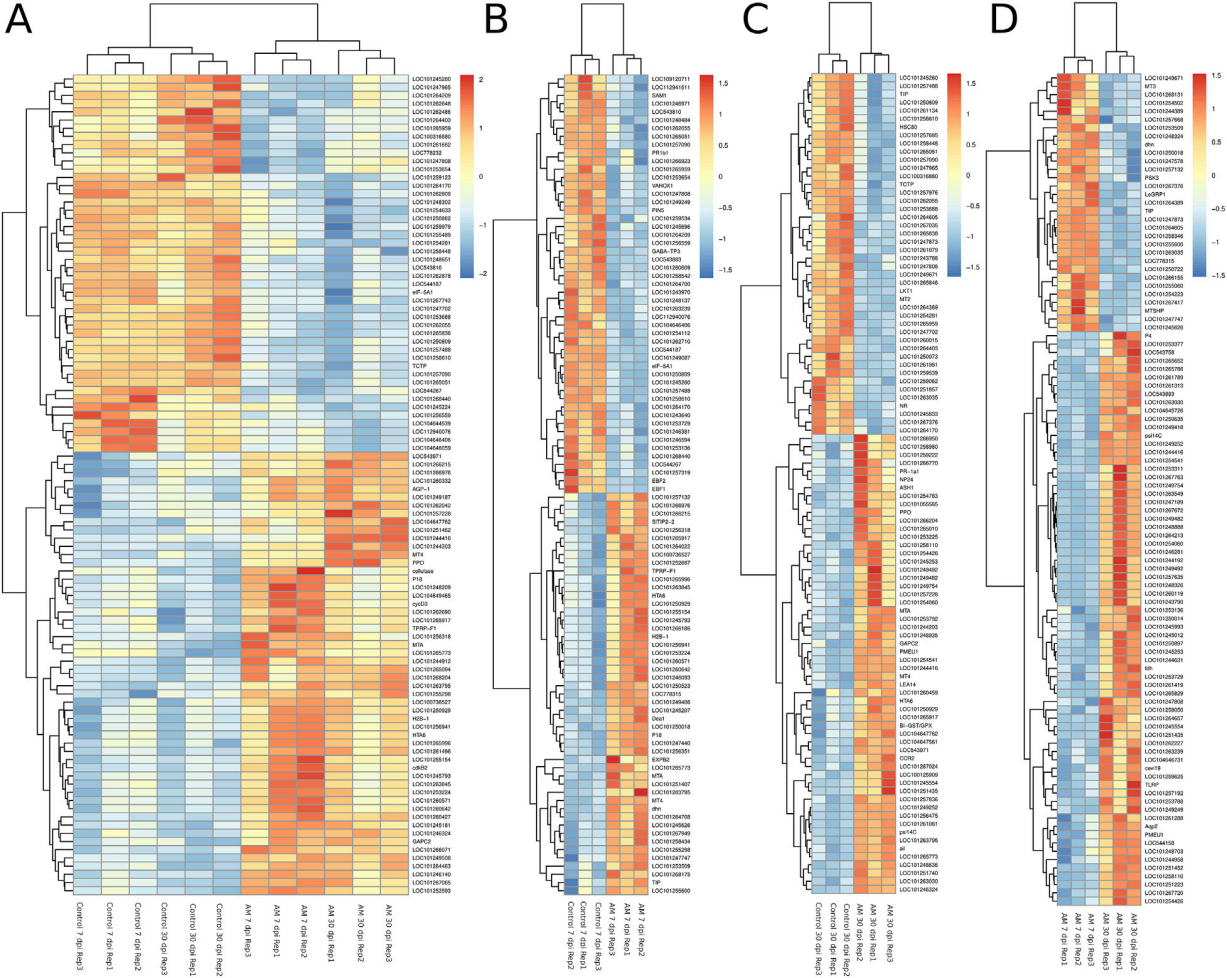
Heatmaps of the differentially expressed genes (DEGs) across various comparisons: **(A)** control 7 and 30 dpi vs. AM 7 and 30 dpi, **(B)** control 7 vs. AM 7 dpi, **(C)** control 30 vs. AM 30 dpi, and **(D)** AM 7 dpi vs. AM 30 dpi.

**FIGURE 3:**
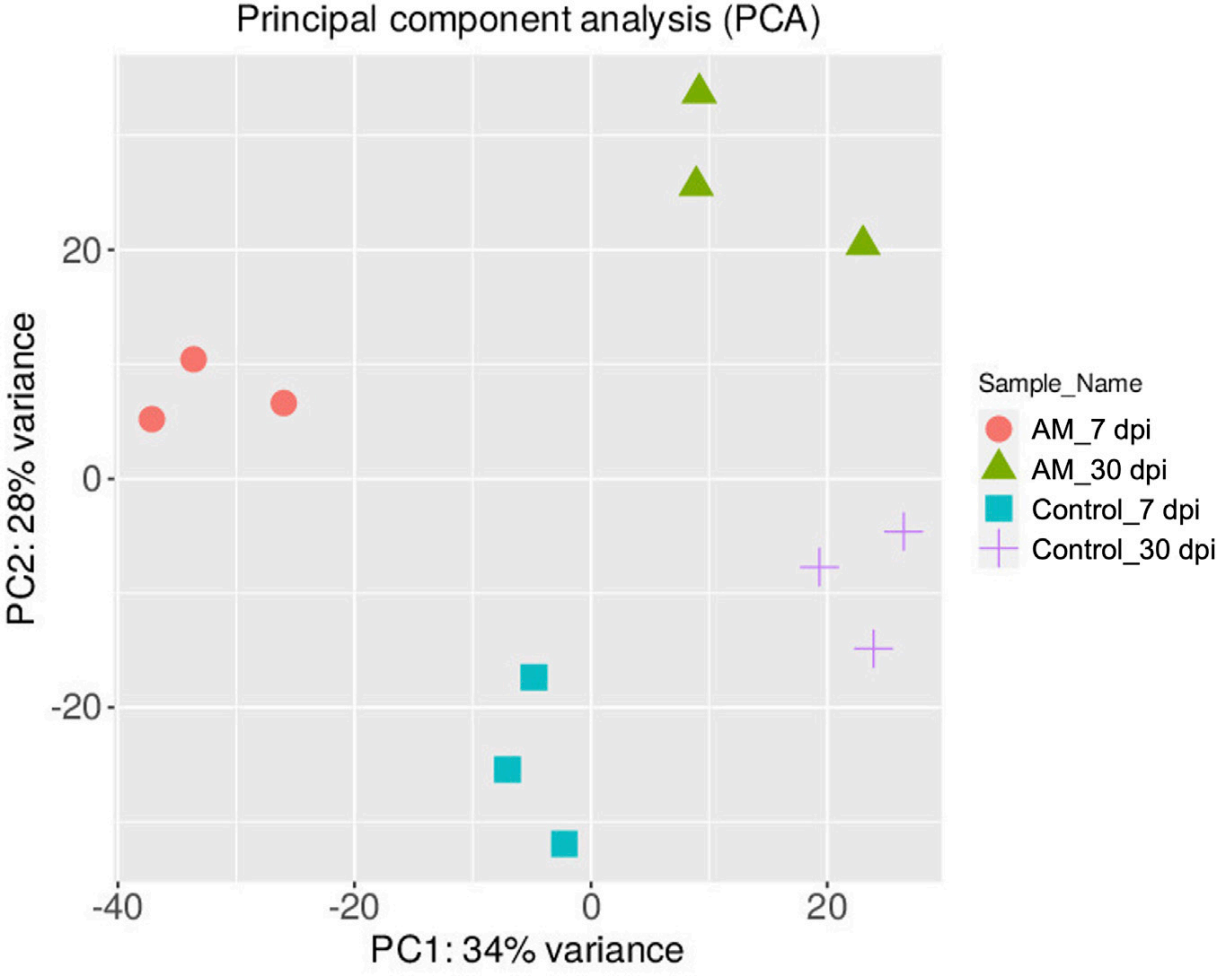
Principal component analysis (PCA) plot of the transcriptome profiles showing the variations based on AM inoculation stages of 7 and 30 dpi.

The DEGs were identified by pairwise comparisons of the libraries obtained from AM-treated and control samples. A total of 1,019 DEGs were identified in the AM-inoculated and non-inoculated control roots at the two time points (AM vs. control), including 635 upregulated and 384 downregulated genes (Figure 2A). In particular, 558 DEGs were detected when comparing the AM-inoculated and non-inoculated roots (control) at 7 dpi (control 7 dpi vs. AM 7 dpi), with 354 upregulated and 204 downregulated genes (Figure 2B). In addition, 320 DEGs were identified in the AM-inoculated and non-inoculated roots (control) at 30 dpi (control 30 dpi vs. AM 30 dpi), with 184 upregulated and 136 downregulated genes (Figure 2C). Lastly, 498 DEGs were found in the AM-inoculated roots when comparing 7 and 30 dpi (AM 7 dpi vs. AM 30 dpi), with 344 upregulated and 154 downregulated genes (Figure 2D). PCA was used to determine the main sources of variance and to identify the inherent structures in the studied phases. The analysis revealed clear clustering, which was particularly noticeable in the control groups at stages I and II and in the AM-treated samples at the same stages. Interestingly, the analysis also showed the ability to differentiate between the control and treatment sublevels within both stage I and stage II samples. PC1 and PC2 together accounted for approximately 62% of the total variance in the dataset, with PC1 explaining 34% and PC2 explaining 28% of the variance, as shown in Figure 3.

**FIGURE 4:**
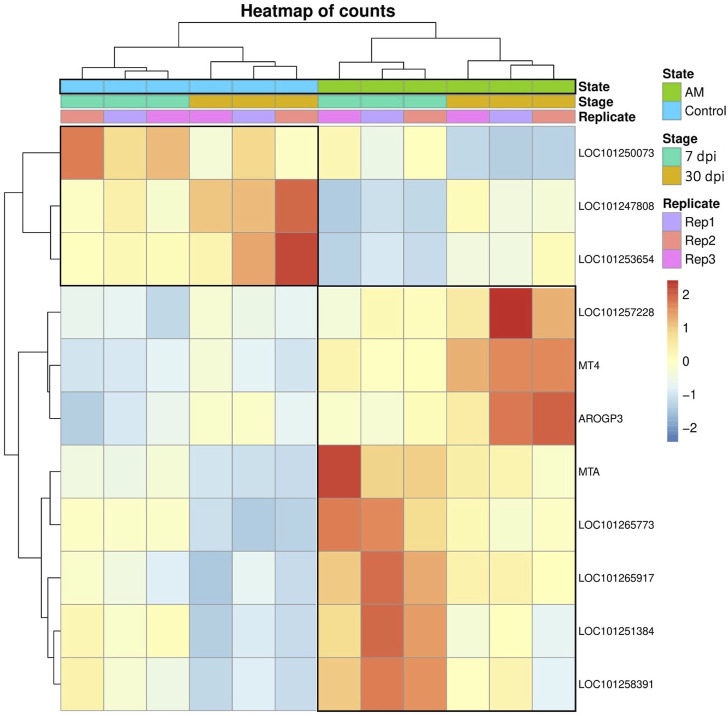
Heatmap analysis of the 11 upregulated gene expressions associated with all conditions.

**FIGURE 5:**
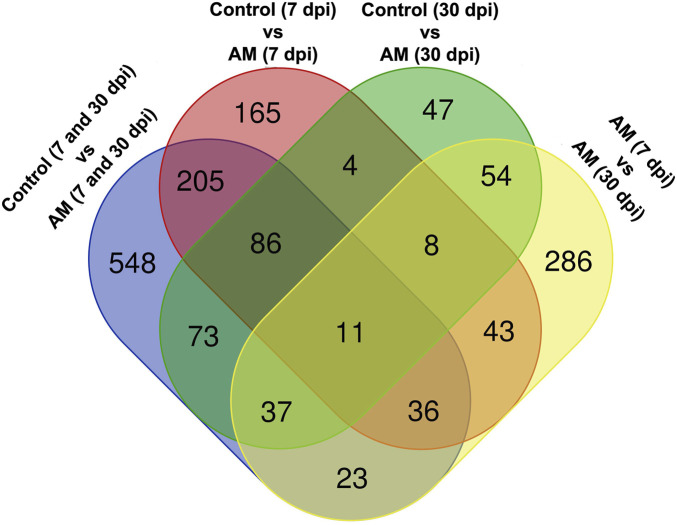
Venn diagram of the DEGs under different comparative groups: AM-inoculated roots at 7 dpi, AM-inoculated roots at 30 dpi, non-inoculated roots (control) at 7 dpi, non-inoculated roots (control) at 30 dpi), AM-inoculated roots at 7 and 30 dpi, and non-inoculated roots (control) at 7 and 30 dpi.

These results suggest that PCA captures the data variability effectively and provides valuable insights into the clustering of samples based on the treatment levels. In addition, the observed clustering supports the results from the differential expression analysis, indicating the ability to distinguish different sample groups by treatment type and stage. The Venn diagram analysis revealed 11 genes common to the AM 7 dpi vs. AM 30 dpi, AM 7 dpi vs. control 7 dpi, AM 30 dpi vs. control 30 dpi, and AM 7 and 30 dpi vs. control 7 and 30 dpi cases. The notable genes include *LOC101247808* (putative late blight resistance protein homolog R1A-3 (*NRC4c*)), metallothionein II- like protein (*MTA*), plant growth and development *LOC101265917* (classical arabinogalactan protein 5 (*AGP5*)), amino acid transporter AVT6A (BP), metallothionein 4 (*MT4*), peroxidase 51 (*POD51*), probable ubiquitin-conjugating enzyme E2 24 (*UBC24*), gamma-aminobutyrate transaminase 2-like (*GABAT*), JA-regulated polygalacturonase non-catalytic subunit (*AROGP3*), and NAD(P)H:quinone oxidoreductase (*NQO1*) (Figures 4, 5).

### 3.3 GO enrichment analysis and PPI network construction

**FIGURE 6:**
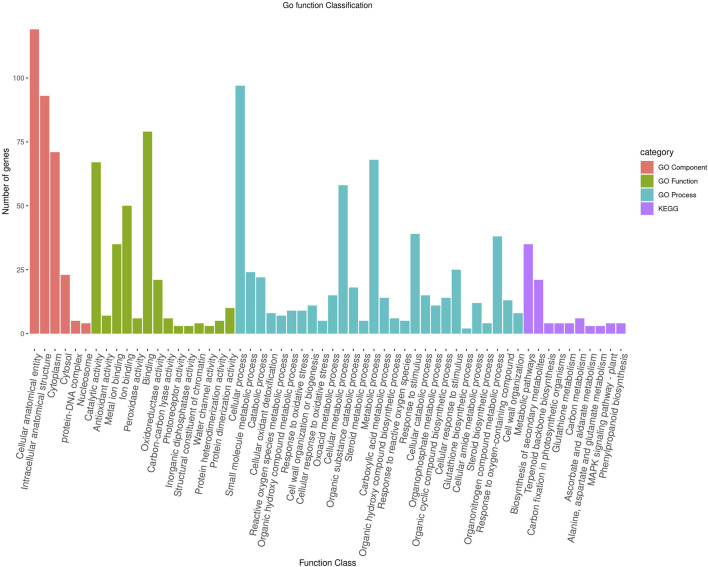
Functional enrichment of the DEGs according to their roles in the biological processes, cellular components, and molecular functions along with their involvement in the KEGG pathways at a significance threshold of FDR >0.05. The figure illustrates the numbers of genes associated with each of the GO terms as a bar graph.

**FIGURE 7:**
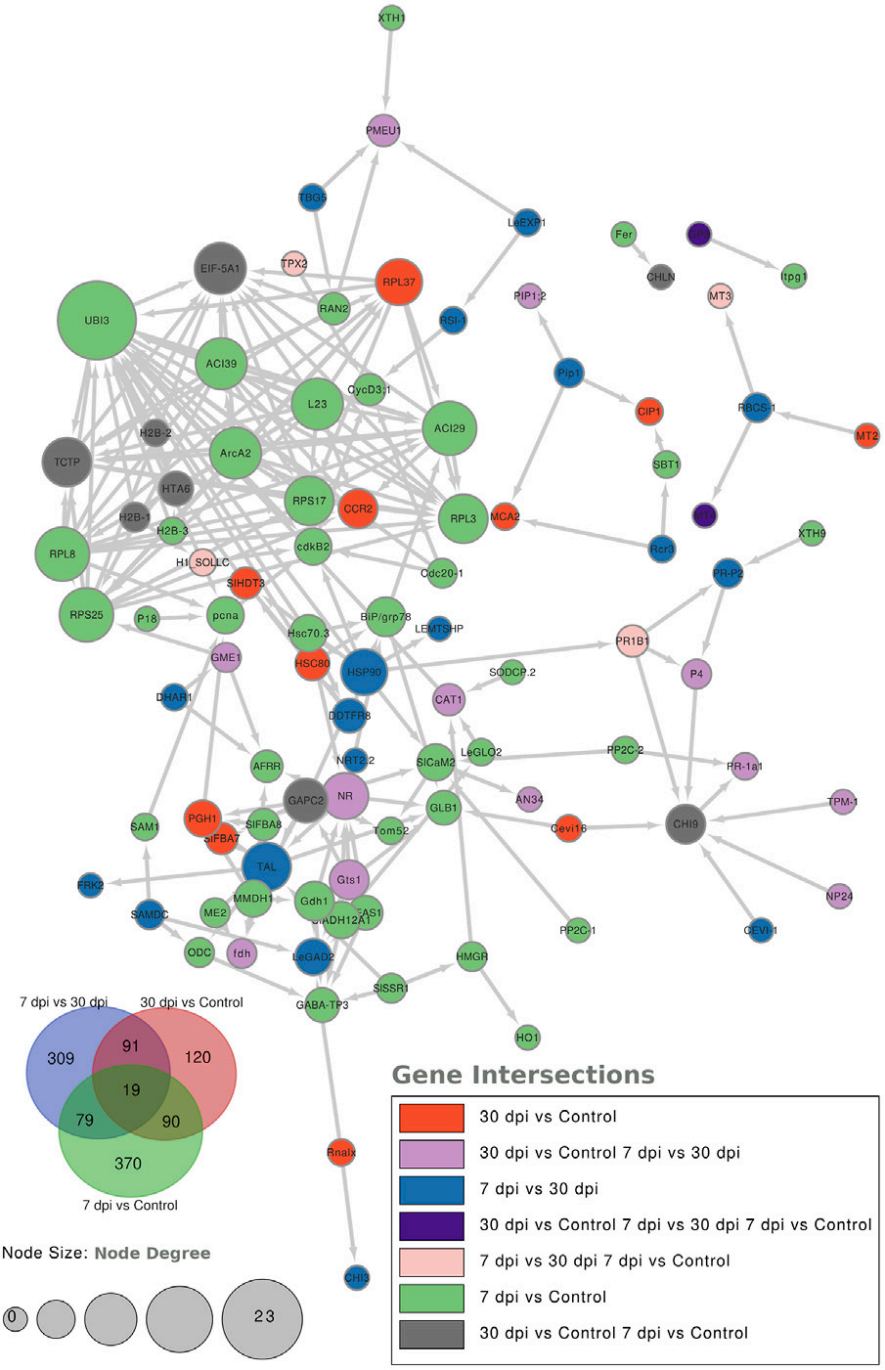
Protein–protein interaction (PPI) network analysis using the STRING database highlights the significantly expressed genes in the comparisons: 7 dpi vs. control, 30 dpi vs. control, and 7 dpi vs. 30 dpi. The analysis shows the interactions between these genes, with the node size representing the node score such that genes with higher interactions have larger node sizes. Additionally, colors are used to indicate the gene categories. For instance, genes uniquely found in the 7 dpi vs. control group are colored blue.

To further characterize the main biological functions of the DEGs in tomato roots under the influence of AMF, the GO functional enrichment analysis was performed. In terms of the functional GO annotations, a total of 1,019 DEGs were classified into 48 functional pathways (FDR ≥ 0.05), including 28 pathways for the BPs, 14 for the MFs, and 6 for the CCs (Figure 6). In the KEGG analysis, the DEGs were annotated into 10 pathways (FDR ≥ 0.05) (Figure 6). The enriched pathways within the BP, CC, MF, and KEGG categories, such as response to oxidative stress (GO:0006979), cellular response to oxidative stress (GO:0034599), response to ROS (GO:0000302), antioxidant activity (GO:0016209), peroxidase activity (GO:0004601), MAPK signaling pathway, and glutathione metabolism, probably play crucial roles in improving the resistance of tomato to abiotic stresses. Using the STRING database, we created a PPI network. After filtering out non-interacting proteins, the PPI network contained 72 nodes and 118 edges for the DEGs. The network highlights significant interactions between several DEGs, with 12 genes that have particularly high interaction rates: monodehydroascorbate reductase (*AFRR*), Actin, ascorbate peroxidase (*APX*), glyceraldehyde-3-phosphate dehydrogenase 2 (*GAPC2*), eukaryotic translation initiation factor 5A1 (*EIF-5A1*), cycloeucalenol cycloisomerase (*CYC1*), cytochrome P450 (*CYP*), GDP-D-mannose 3,5-epimerase (*GME1*), 3-hydroxy-3-methylglutaryl coenzyme A reductase (*HMGR*), calmodulin (*SlCaM2*), cyclin-dependent kinase B (*CDKB2*), and proliferating cell nuclear antigen (*PCNA*) (Figure 7).

## 4 Discussion

The plant root is the central organ for sensing and recognizing symbiotic soil fungi and signaling responses. In this study, we detected major transcriptomic perturbations in tomato roots treated with *R. irregularis* over experimental periods of 7 and 30 dpi. Our results indicate that AM symbiosis induces vital effects in the tomato roots, such as nutrient availability as well as abiotic and biotic stresses (Abarca et al., 2024; Singh et al., 2024; Pellegrino et al., 2024). In this study, RNA-seq experiments were performed on tomato roots after inoculation with *R. irregularis* for a comprehensive overview of the changes in gene expressions. In addition, the molecular mechanisms and signaling pathways underlying the effects of long-term AM interactions with root systems were investigated to provide valuable insights into the symbiotic relationships between tomato roots and AMF. In recent years, several omics technologies have emerged and gained acceptance in disciplines, such as plant sciences and life sciences. Among these technologies, RNA-seq stands out as it enables more precise and comprehensive understanding of the differences in gene expressions between different conditions or phenotypes (Panthee et al., 2024; Zhao et al., 2018). Therefore, a global gene expression study using RNA-seq is essential to better understand the molecular basis of the tomato plant at different developmental stages. Our analysis identified 1,019 DEGs in the roots of the tomato plant; among these, 635 genes were upregulated and 384 were downregulated, suggesting that these genes may play important roles in the symbiotic relationships between tomato and mycorrhiza. To further clarify the functions of these genes in tomato, we subjected the identified DEGs to GO enrichment analysis (Figure 5). The present work identified a set of transcription-associated genes during AMF symbioses in tomato roots colonized at 7 and 30 dpi. These genes are associated with plant defense (*LOC101247808* (*NRC4c*), *MTA*, *LOC101265917* (*AGP5*), *AVT6A* (BP), *MT4*, *POD51*, *UBC24*, *GABAT*, *AROGP3*, and *NQO1*.

*UBC24*, which was identified as PHO2 and negatively regulates Pi uptake, was downregulated at 7 and 30 dpi upon AM inoculation. This could indicate a role of AM in Pi uptake in plant roots and promote resistance to fungal infections (Grennan, 2008; Val-Torregrosa et al., 2022). Although the *AVT6A* gene plays an important role in abiotic stress tolerance (Ayyappan et al., 2024), its expression was downregulated under AM inoculation at 7 and 30 dpi, suggesting that AM has a negative effect on *AVT6A* phosphorylation status in tomato roots (Tahmasebi et al., 2023). The positively affected gene was *AROGP3*, which encodes a JA-regulated polygalacturonase gene with a non-catalytic subunit; it is believed to introduce AM into the plant upon long-term inoculation (30 dpi) by producing aromatic amino acids such as phenylalanine and tyrosine, which are essential for AMF growth and development (Bergey et al., 1999). In addition, the *GABAT* gene is a key enzyme involved in the degradation of γ-aminobutyric acid (GABA) to succinic semialdehyde and is strongly expressed in AM-inoculated roots, especially at 30 dpi. This is considered to positively influence ion transport in tomato plants and consequently plant growth (Ramesh et al., 2015; Hu and Chen, 2020).

Positive regulation of *AGP* in the AM-inoculated roots is thought to play an important role in short-term (7 dpi) inoculation, especially to recognize and attract root colonization by AM-forming mycorrhizae (Nguema-Ona et al., 2013). The *POD* gene is an antioxidant responsible for the removal of H_2_O_2_ and plays a crucial role in various functions. When plant roots are inoculated with AMF, they induce the roots to produce POD, which catalyzes the formation of lignin and oxidative phenolics; this process is essential for plant defense mechanisms and structural support (Song et al., 2010). We hypothesize that *NQO1* plays a role in haustorium development and was positively regulated at 7 and 30 dpi to support the interactions between plant roots and AMF (Bandaranayake et al., 2010). The *MTA* and *MT4* genes were positively expressed under AM inoculation, confirming their roles in the antioxidant functions of the plant in response to exogenous elicitors and antioxidant inducers (Dabrowska et al., 2021). In addition, we hypothesize that the *MT4* gene promotes accumulation of essential minerals and phytoprotective genes in tomato root upon inoculation with AMF (Kısa et al., 2016). In the present work, the PPI network analysis of the altered proteins showed the interaction of a particular protein in the AM-inoculated plants at the two stages (7 and 30 dpi) with 12 other proteins (Figure 7). *AFRR*, *GME1*, and *APX* are involved in the ascorbic acid metabolic pathway, confirming the role of AMF colonization in increasing the ROS levels (Ruiz-Lozano et al., 2012). *CYP*, *GAPC2*, and *CAM2* are involved in metabolic pathways required for root and soil colonization (Mitra et al., 2004; Handa et al., 2015; Keymer et al., 2017). The transcription of genes involved in the sterol pathway, such as *CYC1* and *HMGR*, suggests that AMF enable transfer of lipids from the plant host, which is a hallmark of its obligate biotrophy, as *R. irregularis* does not possess the genes encoding cytosolic fatty acids (Keymer et al., 2017). The observation of the expression patterns of *CDKB2* and *PCNA* as genes related to cell division and cell cycle confirm the roles of AM in supporting plant roots during plant development and stress tolerance (Boyno et al., 2023).

## 5 Conclusion

This study reveals the dynamic transcriptomic changes in the roots of *S. lycopersicum* L. after inoculation with *R. irregularis* and demonstrates the shifts in gene expressions over the short term (7 days) and long term (30 days). Using RNA-seq technology, it was possible to identify numerous DEGs that play crucial roles in improving nutrient uptake, stress resistance, and overall plant vigor. The results imply significant temporal changes in plant defense, growth, and metabolic processes while highlighting the complex molecular interplay between tomato roots and AMF. These insights into phased genetic responses to AM colonization not only deepen our understanding of plant–microbe symbioses but also pave the path for using these interactions to develop sustainable agricultural practices aimed at improving plant resilience and productivity under changing environmental conditions.

## Data Availability

The dataset presented in this study can be found in an online repository. The name of the repository and accession number can be found below: https://www.ncbi.nlm.nih.gov/, PRJNA1151002.
